# What Is the Clinical Relevance of TNF Inhibitor Immunogenicity in the Management of Patients With Rheumatoid Arthritis?

**DOI:** 10.3389/fimmu.2020.00589

**Published:** 2020-04-07

**Authors:** Puja Mehta, Jessica J. Manson

**Affiliations:** Department of Rheumatology, University College London Hospital (UCLH), London, United Kingdom

**Keywords:** immunogenicity, anti-drug antibodies, biopharmaceutical products, TNF-inhibitors, rheumatoid arthritis

## Abstract

Tumor necrosis factor-α inhibitors (TNFis) have revolutionized the management of rheumatoid arthritis (RA), however despite considerable progress, only a small proportion of patients maintain long-term clinical response. Selection of, and switching between, biologics is mainly empirical, experiential, and not evidence-based. Most biopharmaceutical proteins (BP) can induce an immune response against the foreign protein component. Immunogenicity and the development of anti-drug antibodies (ADAs) is considered one of the main reasons for loss of therapeutic efficacy (secondary failure). ADAs may neutralize and/or promote clearance of circulating BP with resultant low serum drug levels, loss of clinical response, poor drug survival and adverse events, such as infusion reactions. ADA identification is technically difficult and not standardized, making interpretation of immunogenicity data from published clinical studies challenging. Trough TNFi drug levels correlate with clinical outcomes, exhibiting a “concentration-response” relationship. Measurement of ADA and drug levels may improve patient care and improve cost-effectiveness of BP use. However, in the absence of clinically-validated, reliable assays and consensus guidelines, therapeutic drug monitoring (TDM) and immunogenicity testing have not been widely adopted in routine clinical practice in Rheumatology. Here we discuss the utility and relevance of TDM and immunogenicity testing of TNFis in RA (focusing on the most widely used TNFis globally, with the most available data, i.e., infliximab, adalimumab, and etanercept), the limitations of currently available assays and potential future immunopharmacological strategies to personalize disease management.

## Introduction

Biologic agents, such as TNF-α inhibitors (TNFis), have revolutionized the treatment of rheumatoid arthritis (RA), but despite this advance, not all patients respond favorably. Up to 40% of RA patients do not respond to the first biologic (primary failure) or lose response over time (secondary failure). Drug survival (the time to discontinuation of a drug) is influenced by many factors including lack or loss of efficacy, adverse events (AEs), and poor adherence. Immunogenicity is defined as the ability of biopharmaceutical products (BPs) to induce an immune response, resulting in the generation of anti-drug antibodies (ADAs). ADAs are considered an important (albeit not the only) mechanism of secondary treatment failure and limited drug survival, due to effects on pharmacokinetics and bioavailability. ADAs are also implicated in treatment-related AEs, such as infusion and injection-site reactions (1). Immunogenicity testing is a mandatory, regulatory requirement for BP drug licensing, as part of the safety profile package required by both the US Food and Drug Administration (2) (FDA) and the European Medicines Agency (3) (EMA).

Therapeutic drug monitoring (TDM) and immunogenicity testing, using trough drug levels and ADAs, have the potential to improve clinical decision-making, by influencing drug selection, dose, and frequency of administration. This may allow clinicians to reduce under- and over- treatment for patients in clinical relapse or remission. There are currently no consensus guidelines recommending the use of BP drug levels and immunogenicity testing in RA, and as such, their use in clinical practice is widely variable.

TNFis (in combination with methotrexate and as monotherapy) are often selected as first-line biologic therapy in patients with RA who are refractory to non-biologic disease-modifying antirheumatic drugs (DMARDs), due to the availability of long-term data from clinical trials and extensive real world experience. Moreover, costs have recently lowered due to the advent of biosimilar TNFis. Infliximab, adalimumab, and etanercept (in bio-original and biosimilar forms) are the most frequently used TNFis, with the most available data. Here, we discuss the utility and clinical relevance of TDM and immunogenicity testing of TNFis in patients with RA, and potential future immunopharmacological strategies to personalize disease management.

## Immunogenicity of TNFis in RA

### Consequences of Immunogenicity

Immunogenicity can impact both the efficacy and safety of BPs. ADAs may reduce the clinical efficacy of TNFis by competing with the cytokine binding site (neutralizing antibodies) or by accelerating drug clearance leading to subtherapeutic drug levels (non-neutralizing/binding antibodies; with formation of immune complexes), hence both neutralizing and non-neutralizing ADAs may be clinically relevant. Trough TNFi drug levels exhibit a “concentration-response” relationship (4) (an inverse correlation with clinical outcomes), which forms the basis for the rationale for TDM in RA. This has been observed in studies of the key TNFis used in clinical practice—including infliximab (5–10), adalimumab (11), etanercept (12, 13), golimumab (14), and certolizumab (15, 16).

ADAs are associated with low trough drug levels and loss of drug efficacy, although the association appears to be stronger for infliximab, adalimumab, and golimumab, than for etanercept and certolizumab (4). ADAs in isolation do not always correlate with poor clinical outcomes, as the antibody titer may be insufficient to reduce the active drug level below the therapeutic threshold. Furthermore, the risk of immunogenicity is not sufficient to predict loss of drug efficacy e.g., although adalimumab is more immunogenic than etanercept, some studies report only a small difference in drug survival (17, 18).

ADAs have been linked to several AEs including infusion/injection site hypersensitivity reactions, serum sickness, and arthus reactions (1, 19). The pathogenic mechanisms are yet to be fully elucidated and may involve complement-mediated events, cytokine release, formation of immune complexes, and production of IgE antibodies. Reassuringly, switching from bio-original to biosimilar BP, has not been associated with greater AEs or immunogenicity concerns thus far (20).

### Factors Influencing Immunogenicity

Historically, the foreign (murine) components of the drug were thought to be mainly culpable for the development of ADAs, which led to a drive to minimize non-human elements to reduce immunogenicity. It soon became apparent that even fully human BPs could provoke an immune response, due to TNF-binding idiotypes that are not part of the normal human antibody repertoire, and multiple factors influencing immunogenicity are now emerging. TNFis may be chimeric (e.g., infliximab), humanized (e.g., certolizumab), fully human (e.g., adalimumab and golimumab), or fusion proteins containing antibody fragments (e.g., etanercept). Infliximab is considered the most immunogenic TNFi, particularly when it is used without concomitant methotrexate (21, 22). ADAs have been reported in up to 53% of patients treated with infliximab within the first 6 months of treatment (5, 8, 23–25). By contrast, in the same timeframe, up to 19% of patients receiving adalimumab develop ADAs (8, 24, 26). Etanercept, a receptor construct, does not express idiotypes and thus is the least immunogenic out of the three; ADAs to etanercept are minimal, usually transient and non-neutralizing with a reported incidence of 0–7% (21, 27, 28).

Effective detection of ADAs is dependent upon several factors—the type of the assay used, the timing of the blood sample in relation to drug dosing (usually trough levels, taken before a scheduled dose) and the duration of treatment. In addition, assay results are affected by the relative amount of drug and antibody: excess serum drug levels can prevent the detection of free ADAs; equal drug and antibody levels can prevent measurement of both; and excess ADA usually permits only the detection of free antibodies (29).

Mechanisms leading to immunogenicity are complex and multifactorial; related to the drug (e.g., purity and aggregations) and its production process (e.g., contaminants), the patient and treatment (1, 30). Patient-related factors include genetic predisposition (31), disease activity (32), obesity (32), smoking (32), and indication (33) for biologic treatment. It is tempting to speculate that ADAs are more likely to be evoked in classical autoimmune diseases, where B-lymphocytes are implicated in disease pathogenesis, e.g., a trend toward higher frequency of ADAs is found in patients with RA compared with psoriasis, when treated with the same biologic (4). However, ADAs are clinically relevant in non-antibody-mediated rheumatic conditions e.g., axial spondyloarthritis (34) and are extensively described in inflammatory bowel disease (IBD). This concept was exemplified in a study of patients with spondyloarthritis (*n* = 294) and rheumatoid arthritis (*n* = 276) with secondary TNFi failure, where significantly more patients with spondyloarthritis (31.3%) had anti-infliximab antibodies, compared with those that had RA (21.1%; *p* = 0.014) (33). Treatment-related factors include the dose, frequency, route, and continuity of administration, prior drug exposures as well as concomitant immunomodulators (35). In general higher doses of the BP or a loading regimen (36) followed by continuous rather than episodic dosing (37), the intravenous (compared with subcutaneous) (38, 39) route of administration and concomitant immunosuppression (28, 40) are associated with a lower frequency of ADAs. However, there are some caveats—subcutaneous delivery (relatively more immunogenic and usually the preferred route of administration for most BPs) of tocilizumab (an anti-interleukin (IL)-6 receptor monoclonal antibody) is not more immunogenic than its intravenous administration (41) and whilst concomitant immunosuppressants reduce immunogenicity in RA and Crohns disease (28, 40), evidence for this strategy is not valid across all indications e.g., methotrexate co-prescription does not significantly influence drug survival of TNFis in psoriatic arthritis populations (42).

### Limitations of Immunogenicity Testing

The clinical application and interpretation of immunogenicity data is challenging as studies of TNFis show wide variation in the prevalence of ADAs, as well as their impact on serum drug concentrations and clinical outcomes. These observations may be due to heterogeneous patient populations and differences in study design, duration of follow-up, drug dosage, use of concurrent DMARDs and timing of blood sampling. Comparisons between publications are difficult due to inter-laboratory variability and inconsistent (and occasionally absent) reporting of assay methods and characteristics. Furthermore, it is very difficult to make comparisons between different assays for different BPs, due to the reliance of each method on the specific positive control used (43).

Even if detection methods are reliable, most available assays do not evaluate the *in vivo* functionality of drug and ADAs, i.e., the amount of active circulating drug or the neutralizing capability of the ADA, which could limit the clinical application of the results.

ADA detection involves either a bridging ELISA (most commonly), or a radioimmunoassay (RIA). Available RAIs include the antigen binding test (radiolabelled therapeutic TNFi antibodies bind to free ADAs in serum samples) or pulldown assays (ADAs are coupled to a high-capacity solid substrate). Both ELISAs and RIAs are only able to detect free ADAs; therefore, high drug levels, with formation of ADA-drug complexes, can lead to false negative results. This is known as “drug interference/tolerance,” where ADAs are only detected if their amount exceeds the level of the circulating drug. ELISAs can further underestimate the presence of ADAs, as they do not identify IgG4 ADAs [which are more likely to be neutralizing (44)] and are less drug-tolerant than RIAs. RIAs are more specific than bridging ELISA, are less prone to interference by drug and rheumatoid factor and can capture clinically relevant IgG1 and IgG4 ADA. RIAs are more sensitive than ELISAs when using random blood samples [with better concordance between the assays when ADA titres are high (45)], which would be more convenient for patients, however their widespread use is limited by the cost and complexity associated with radioisotopes.

From a practical perspective, TDM and immunogenicity testing can be difficult. Ease of access to tests is variable, and it may be difficult to obtain accurately timed blood samples for trough drug levels. Newer drug-tolerant assays that measure both free and complexed ADAs, including the pH-shift anti-idiotype binding tests (PIA), may be more suited to random blood sampling, but these tests are expensive, may only be available in specialized centers and have as yet, undetermined clinical utility (46).

## Current Clinical Practice

Current options for managing TNFi failures in RA include cycling within class, i.e., to an alternative TNFi, or switching between class i.e., to a drug with a different mechanism of action. Published recommendations provide little guidance to determine the best strategy (47, 48). Both options are supported by data from randomized controlled trials and the real world, therefore the decision is generally empirical and based on physician discretion. This dilemma was summarized in a recent review (29). In the open-label, 52 weeks randomized Rotation or Change (ROC) trial, the treating physician selected between a second TNFi and a non-TNFi in patients with primary TNFi failure (49). The ROC trial results concluded that the reasons for improved drug survival when switching to a second TNFi was better efficacy, and with switching to a non-TNFi was reduced AEs. Further evidence from a prospective study, suggests better outcomes can be achieved using an algorithm based on trough drug levels and ADAs, compared with “empirical switching” (50).

Current treatment recommendations for RA endorse combination therapy with a biologic and DMARD (47, 48), which is consistently more effective than biologic monotherapy, possibly due to effects on immunogenicity. Methotrexate significantly increases adalimumab trough concentrations (51, 52), and in a dose- dependent manner, reduces immunogenicity (51), and improves clinical outcomes in early disease (53).

Given the limitations regarding assay diversity and data interpretation, and the lack of conclusive support for cost- effectiveness, routine use of TDM and ADA testing has not been widely adopted in British Rheumatology practice (54). There are exceptions, with local management algorithms for RA incorporating these tests (55, 56), but overall the use and interpretation of TDM and ADAs is inconsistent. By contrast, The British Society for Gastroenterology guidelines for the management of IBD includes clear, algorithmic recommendations for measurement of drug levels (±ADA) (57). In IBD, clinical decision making using drug levels and ADAs in secondary non-responders is more cost-effective when compared to empirical drug escalation (58, 59). The recent prospective, observational personalized anti-TNF therapy in Crohn's disease study (PANTS), demonstrated that low concentrations of adalimumab and infliximab at week 14 were associated with primary non-response, non-remission at week 54 and the development of ADAs (32). ADAs predicted subsequent low drug levels and concomitant immunomodulators (thiopurine or methotrexate) mitigated the risk of developing ADAs (32).

## Potential Immunopharmacological Algorithm

**Figure 1:**
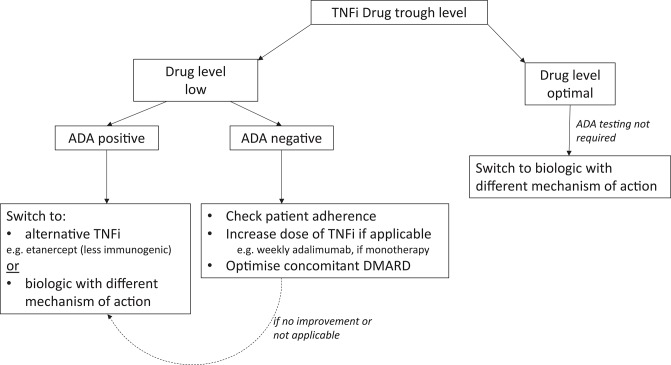
Potential algorithm for RA patients with secondary failure to TNFis. ADA, Anti-drug antibody; BP, Biopharmaceutical product; TNFi, Tumor Necrosis Factor inhibitor.

In time, readily available, accurate assays to measure drug levels and ADA titer, will hopefully arm clinicians with powerful tools to optimize the management of RA, especially in patients with secondary loss of response. A potential algorithm that could be used in future management strategies is shown in Figure 1.

Measurement of trough drug levels is the most valuable test in the first instance to identify patients with low or optimal (therapeutic) circulating drug. Using ADAs for the first branching in the algorithm is probably inappropriate, as ADAs are not always clinically relevant (especially if present at low-titer) if there is sufficient circulating drug. In cases of treatment failure, supplementary knowledge of ADAs (and perhaps the titer) may be helpful in determining the etiology of suboptimal drug levels. Low drug levels without ADAs may be due to factors such poor adherence to therapy (as most biologics are self-administered injections), a higher BMI and/or faster drug metabolism, which would require different strategies compared with those for patients with detectable ADAs. To overcome this problem, optimizing the dose of biologic by reducing the interval of administration, e.g., changing adalimumab monotherapy from fortnightly to weekly [as permitted by the National Institute of Health and Care Excellence (NICE) in the U.K. (60)], or optimizing dose of concomitant immunosuppressants may recapture a response (61, 62). Emerging evidence suggests that efficacy can be re-established in ADA positive patients with secondary failure, by addition of methotrexate to infliximab treatment in IBD (63), although there is limited support for this approach in the RA literature. If these strategies are unsuccessful or not applicable, switching BP should be considered.

If ADAs are detected in the context of a low drug level, switching to a less immunogenic drug within the same class (e.g., etanercept) could be beneficial, especially if the patient has previously responded to a TNFi. Switching to a second TNFi may be successful due to differences in drug molecular structure, immunological action, immunogenicity, and pharmacokinetics, as well as different underlying disease pathogenesis (24). There is an argument however, to switch to a biologic with a different mechanism of action, as although ADAs are not cross-reactive, patients with ADAs to the first failed TNFi are more likely to seroconvert and produce ADAs with subsequent TNFis (64–67) and are thus less likely to respond to a second TNFi, especially if this is a monoclonal antibody (64, 66). Of note, ADAs to bio-originals are reactive to the corresponding biosimilars, and therefore after detection of ADA, switching a bio-original to its biosimilar version would not be recommended (68). It is plausible to suggest that a patient with ADAs, refractory to multiple biologics, may benefit from a treatment with a less or minimally immunogenic drug, e.g., a receptor fusion protein e.g., abatacept (69) or a small molecule [JAK inhibitor (70)]. In the case of non-responders with optimal drug levels, the presence/absence of ADAs is unlikely to influence subsequent management. These patients have a lower probability of response to an agent within the same class and therefore we would postulate that they are most likely to benefit from switching to a drug with a different mechanism of action (64).

Given the high cost and potential AEs associated with biologic therapies, strategies have been proposed to taper biologics (by reducing drug doses or increasing dosing intervals) in patients with sustained clinical remission, thereby reducing risks and costs overtreatment. In some studies, correlation between DAS28 (disease activity score; a composite measure of disease activity in RA) improvement and serum drug trough levels has been verified up to a threshold of drug level, above which no significant DAS28 changes occur (71). A recent study using certolizumab found that a drug level above a defined threshold was not associated with any additional clinical benefit, and therefore it may be possible in the future to use TDM to titrate treatment (15). Withdrawal of treatment in disease quiescence is an area of active research and currently there is insufficient evidence to draw meaningful conclusions about the role of TDM and immunogenicity testing. Data from ongoing, randomized controlled trials (72) using TDM or ADA to guide withdrawal strategies may inform future practice. It is reasonable to hypothesize that drug withdrawal may be possible in patients with inactive disease and undetectable drug levels or high ADA titres, as remission is probably not being maintained by treatment with the BP.

## Future Directions and Unanswered Questions

The increasing and earlier use of BPs in RA is likely to lead to a greater proportion of patients receiving these therapies. Efforts are expanding to predict, reduce and reverse BP immunogenicity to mitigate the impact on drug development, which was summarized in a recent review (73). Strategies to reduce the immunogenic potential of BPs include “de-immunizing” approaches through protein engineering e.g., rational amino acid substitutions and/or addition of epitope-masking moieties, as well as induction of peripheral tolerance (73). There are emerging concerns that immunogenicity may limit the development of newer investigational medicinal products such as the bispecific antibodies.

Despite long-standing interest and accrual of data, we are still unable to predict responses to TNFi. Prospective, longitudinal studies of BP-naïve patients may provide mechanistic information and address a critical unanswered question—why BPs are immunogenic in some patients, but tolerogenic in others. Prediction of immunogenicity may allow mitigation and management strategies to be implemented to prevent or minimize the generation of ADAs (73). Other strategies to personalize biologic selection, include pharmacogenetic testing to identify genetic factors that may predict lack of response to, or toxicities from, TNFi (74).

Further research is needed to develop standardized, clinically-validated assays for both drug and ADA testing. These tests could then be incorporated into evidence-based guidelines to optimize treatment decisions along the patient pathway: for patients with active disease about to start treatment, not responding to treatment (primary or secondary failure) or for those in remission, to permit drug tapering strategies. Taken together this may help to improve the long-term efficacy, safety profile and cost-effectiveness of BPs.

## Author Contributions

PM and JM co-wrote this manuscript and both authors approved the final version.

### Conflict of Interest

The authors declare that the research was conducted in the absence of any commercial or financial relationships that could be construed as a potential conflict of interest.
